# Pars Interarticularis Fractures Treated with Minimally Invasive Surgery: A Literature Review

**DOI:** 10.3390/jcm13020581

**Published:** 2024-01-19

**Authors:** Adrienne Minor, Benjamin R. Klein, Mareshah N. Sowah, Kayla Etienne, Allan D. Levi

**Affiliations:** 1 School of Medicine Greenville, University of South Carolina, Greenville, SC 29605, USA; am249@email.sc.edu (A.M.); msowah@email.sc.edu (M.N.S.); 2 Department of Neurological Surgery, Miller School of Medicine, University of Miami, Miami, FL 33146, USA; benjamin.klein@rockets.utoledo.edu (B.R.K.); kayla.etienne@tufts.edu (K.E.); 3 College of Medicine and Life Sciences, University of Toledo, Toledo, OH 43560, USA; 4 School of Medicine, Tufts University, Boston, MA 02108, USA

**Keywords:** minimally invasive, pars interarticularis, percutaneous pedicle screws

## Abstract

Recurrent stress on the isthmic pars interarticularis often leads to profound injury and symptom burden. When conservative and medical management fail, there are various operative interventions that can be used. The current review details the common clinical presentation and treatment of pars injury, with a special focus on the emerging minimally invasive procedures used in isthmic pars interarticularis repair. PubMed and Google Scholar database literature reviews were conducted. The keywords and phrases that were searched include but were not limited to; “history of spondylolysis”, “pars interarticularis”, “pars defect”, “conventional surgical repair of pars”, and “minimally invasive repair of pars”. The natural history, conventional presentation, etiology, risk factors, and management of pars interarticularis injury are discussed by the authors. The surgical interventions described include the Buck’s repair, Morscher Screw-Hook repair, Scott’s Wiring technique, and additional pedicle screw-based repairs. Minimally invasive techniques are also reviewed, including the Levi technique. Surgical intervention has been proven to be safe and effective in managing pars interarticularis fractures. However, minimally invasive techniques often provide additional benefit to patients such as reducing damage of surrounding structures, decreasing postoperative pain, and limiting the time away from sports and other activities.

## 1. Introduction

The pars interarticularis, also known as the isthmus, lies in between the superior and inferior articular facet joints in the spine [1]. The area is commonly subjected to stress fractures that affect the lower lumbar vertebrae [2]. Spondylolysis refers to a fracture of the pars interarticularis, occurring either bilaterally or unilaterally, and most commonly involves the L4 and L5 pars segments [3,4]. Interestingly, congenital spondylolysis occurs due to weakening of the neural arch during early fetal development and differs in pathogenesis compared to that of the acute traumatic and pars elongation subtypes of spondylolysis [5]. Furthermore, due to the coronal orientation of the L5/S1 facet joint, the L5 pars interarticularis endures a great amount of stress and is a frequent location of injury [5]. Spondylolysis is a relatively common lower back defect, impacting 6–11.5% of the world’s population [6]. The fracture is especially prevalent in athletics, with 7–8% of those affected being young athletes [7]. Athletes such as soccer players, weight-lifters, divers, and gymnasts commonly hyperextend upon rotation which precipitates the injury [8]. Symptomatic patients can experience mechanical lower back pain that worsens with activity and with extension of the spine, as well as tightness of the hamstrings [9]. X-rays are often the initial imaging modalities obtained, but there are a variety of studies such as computed tomography scan (CT), magnetic resonance imaging (MRI), bone scintigraphy, and single-photon emission computed tomography (SPECT) that can aid in diagnosis [10]. In terms of treatment, there are several surgical techniques available to patients who have failed conservative medical management [5]. Minimally invasive surgical techniques have proven to minimize adjacent soft tissue damage, decrease postoperative pain, increase patient satisfaction, and limit the amount of time away from work and/or competitive sports [5]. In this review, we explore the clinical presentation and historical treatment of pars interarticularis fractures in the population of young athletes. We reviewed the current literature to provide information about conventional surgical techniques and emerging minimally invasive procedures that have been proven to be effective in the repair of isthmic pars interarticularis fractures.

### 1.1. Etiology and Risk Factors

The exact pathogenesis of spondylolysis remains unknown. Multiple studies have investigated the families of individuals with spondylolysis, providing evidence that suggests a genetic component to the development of this condition [11,12,13]. Roche and Rowe found significant variations in the incidence of spondylolysis on the basis of ethnicity and sex. Of the 4200 skeletons examined, 4.2% were determined to have a neural-arch separation. Among the skeletons that demonstrated a neural-arch separation, 6.4% were Caucasian males, 2.8% African American males, 2.3% Caucasian females, and 1.1% African American females [14]. Further evidence of sex as a contributing factor in the etiology of spondylolysis was seen through investigation by Tatsumura et al., revealing a significant difference in incidence of spondylolysis among male and female participants. The sex ratio of participants with spondylolysis was 215 (80.2%) boys to 53 (19.8%) girls [15]. The most common etiology of spondylosis is micro-trauma resulting from repetitive mechanical stress to locations of weakness within the vertebrae [16]. There is little to no evidence of spondylolysis in non-ambulatory patients or newborns, supporting the likelihood of mechanical stress as a major contributing factor [4,17].

### 1.2. Natural History

Spondylolysis is derived from the Greek spondylo meaning “spine” and lysis meaning “dissolve” [18]. 6% of archeological skeletons recovered from Romano-British remains had the defect, and up to 20% of Native American populations from 10,000 to 6000 B.C [18]. This highlights the historical nature of this ailment, demonstrating its presence for thousands of years. Spondylolysis was first described by a surgeon from Cambridge named Dr. George Murray Humphry in 1858 [18,19]. Later, the German Prof. Robert zu Coblenz described the importance of a strong pars interarticularis in preventing vertebral olisthesis [19]. Finally, Dr. Franz Neugebauer from Warsaw published the first case describing the direct link between a pars interarticularis defect and erect human posture in 1884 [20]. Many species experience spondylolysis, but only humans experience the defect in the lumbar spine [21]. Since lumbar spondylolysis has not been identified in other primates, it has been speculated that there is congenital weakening of the pars in humans [22].

### 1.3. Clinical Presentation

The presentation of spondylolysis can vary, although most patients are asymptomatic [4,23]. Lisbon et. al. demonstrated that patients experiencing unilateral spondylolysis are less likely to be symptomatic compared to patients suffering from bilateral spondylolysis [24]. While lower back pain that is exacerbated by activity is most commonly seen, symptomatology worsens based on the extent of the defect. It has been postulated that pain from spondylolysis is due to slippage of the vertebral body [16]. These patients are usually evaluated in an outpatient setting for dull and insidious low back pain that has been present for some months [5]. The dull pain can intermittently radiate to the buttocks and thighs and is characteristically described as sharp and/or stabbing [5]. Patients typically present between youth and adolescence, with males being predominantly affected [5]. There is frequently a history of participation in competitive sports such as football, gymnastics, diving, or track and field [7,8,25]. To reduce the differential diagnosis, young athletes experiencing symptoms of spondylolysis should be asked about hyperextensive movements involving the lumbar spine [5,8,18].

### 1.4. Physical Examination

A physical and neurological examination should be performed in all patients presenting with the aforementioned symptoms. A neurological examination suggestive of spondylolysis would reveal reduction in trunk range of motion with extension, increased lumbar lordosis, tight hamstrings, and tenderness to palpation over the site of the pars fracture. A characteristic finding is the absence of radiculopathy [26]. The most diagnostic physical exam finding is the Stork (also known as Gillet) Test, which would demonstrate pain during single-leg hyperextension and spinal rotation, if positive. Physical exam findings may be relatively nonspecific, thus additional evaluation via imaging is appropriate in patients with a suspected pars defect [1].

### 1.5. Imaging

**Figure 1:**
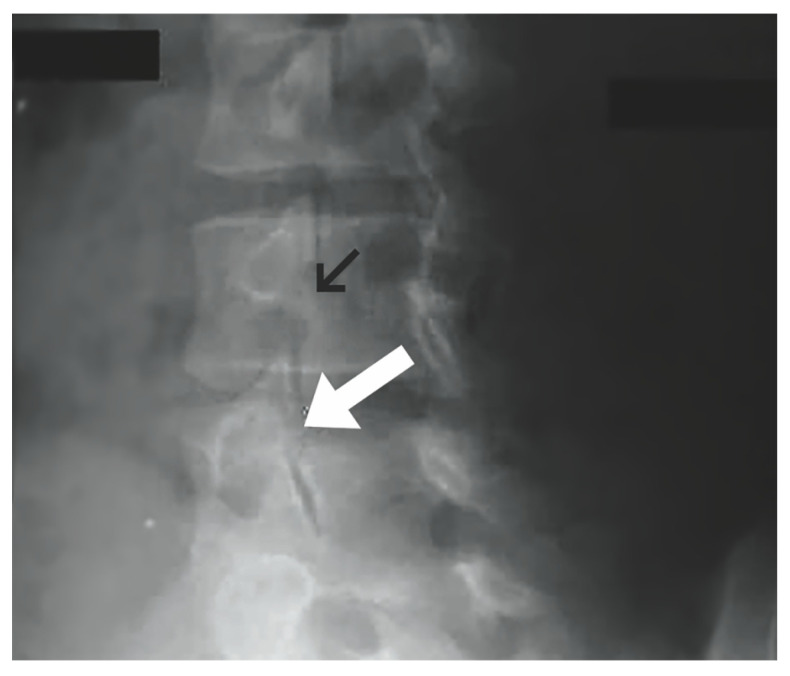
Oblique plain lumbar radiograph. Normal pars interarticularis denoted by black arrow. Pars interarticularis defect with “Scottie dog” sign demonstrated by white arrow. (“Contributed by J Taylor Mansfield, DO” [1,33]) “Reproduced with permission from Mansfield, J.T.; Wroten, M. Pars Interarticularis Defect; published by StatPearls Publishing: Treasure Island, FL, USA, 2023”.

**Figure 2:**
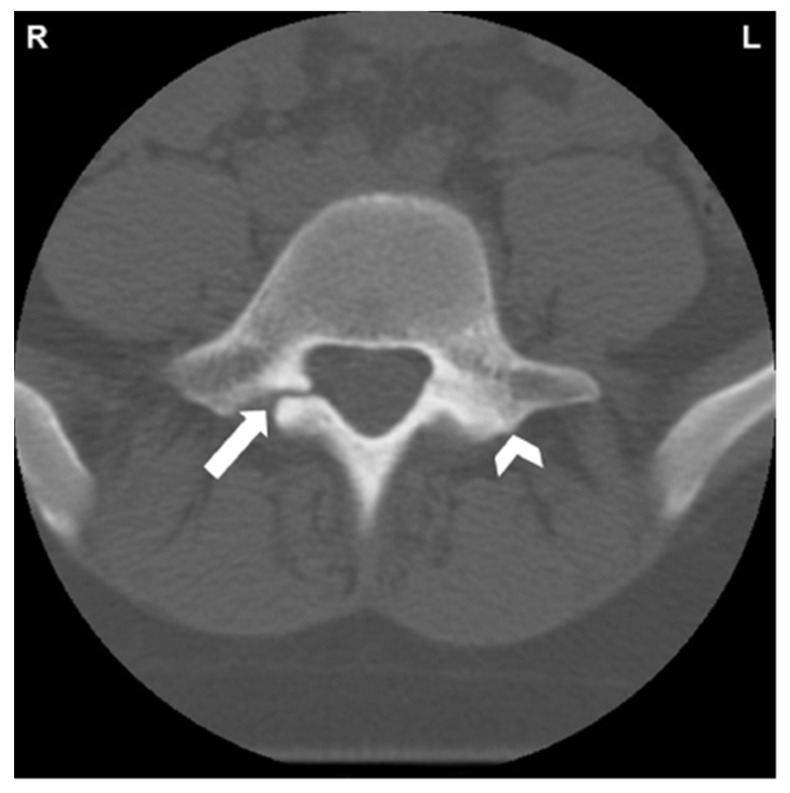
CT of L5 vertebral body and posterior elements. White arrow indicates spondylolysis of right pars interarticularis. Reactive sclerosis in the left pars demonstrated by white arrowhead [34]. “Reprinted from Neural arch bone marrow edema and spondylolysis in adolescent cheerleaders: A case series. *J. Chiropr. Med.* **2019**, *18*, 335–342; with permission from Elsevier”.

Radiographic examination is essential to accurately diagnose pars interarticularis defects. Workup for spondylolysis should begin with plain lumbar radiographs in the anterior-posterior (AP), lateral, or oblique view while in the standing position. A pars interarticularis fracture has classically been identified in the oblique view as the “Scottie dog” sign (Figure 1). Radiation exposure should be considered when evaluating high-risk individuals, such as children and adolescents [1]. The cost effectiveness, availability, and low radiation exposure by plain radiographs make them an ideal initial step. Further evaluation with advanced imaging such as CT, SPECT, and MRI should be carried out if a diagnosis is not evident with plain films [27]. CT has high sensitivity for detection of pars defects and thus may be utilized for diagnosing and staging (Figure 2). Thin-cut CT scans reveal the degree of cortical disruption, lysis and sclerosis, making them the best option for assessing the progression and healing of pars interarticularis defects [28,29]. SPECT is another modality with improved sensitivity that is used to detect stress reactions and acute lesions that may not show on CT or plain radiographs. This nuclear imaging technique is also helpful in evaluating the healing of pars defects [27]. MRI can show bone marrow edema on T2 weighted sequences, which is helpful in distinguishing acute stress fractures from chronic non-union. Additional findings on MRI such as nerve root compression and widened spinal cord sagittal diameter contribute to the overall benefits of this imaging option [29,30,31]. Meyerding classification for spondylolisthesis categorizes the degree of slippage by quantifying the percentage of subluxation of the vertebral body above and below [32].

### 1.6. Non-Operative Management

In athletes, pars fractures caused by repetitive stress have shown the ability to heal with early rest and immobilization [35]. Targeted physical therapy is indicated in spondylolysis, after a period of rest and activity modification, with exercises focusing on deep abdominal musculature and lumbar multifidi [35]. This approach has been proven to alleviate symptoms and improve functional outcomes [35]. The exact time to introduce physical therapy in patients with pars defects has been a point of controversy. Selhorst et. al. performed a retrospective analysis of adolescents that were placed in physical therapy with either less than 10 weeks of rest or more than 10 weeks of rest [36]. The results showed better patient outcomes correlating with earlier introduction to physical therapy [36]. The use of hard-shell bracing as well as thoracolumbosacral and lumbosacral orthotics have been shown to alleviate pain and contribute to positive patient outcomes [35]. A retrospective review by Choi et al. investigated the optimal treatment algorithm for symptomatic spondylolysis in adolescent athletes. A total of 201 adolescent athlete patients diagnosed with spondylolysis were treated conservatively with cessation of sports activity, thoracolumbosacral orthosis, and external bone stimulator for three months. Each patient subsequently received six weeks of rehabilitation. A significantly higher rate of bone healing was identified by follow-up CT scans in 152 athletes who reported using bone stimulators as prescribed compared to those who did not use bone stimulators. A total of 197 patients (98%) returned to sports or similar level of activities and 37 patients (18%) received steroid injections for continued pain. One patient received surgical treatment. Follow-up CT scans revealed bony healing in 49.8% of patients [16].

### 1.7. Follow-Up Evaluation

Once the back pain has subsided or when the patient has completed six months of physical therapy, repeat CT imaging should be performed to ensure that osseous healing, not fibrous, has occurred across the defect [5]. Nuclear imaging studies can also be performed to detect any additional radiotracer uptake at the site of the defect [5].

### 1.8. Surgical Repair Indications 

Surgical intervention is recommended for patients who still experience symptoms of spondylolysis for a minimum of six months despite therapy and other non-operative techniques [5]. Surgery is also recommended for patients experiencing various neurological deficits such as weakness, bowel/bladder incontinence, and high-grade spondylolisthesis [5].

### 1.9. Conventional Surgical Repair Techniques

#### 1.9.1. Buck’s Repair 

**Figure 3:**
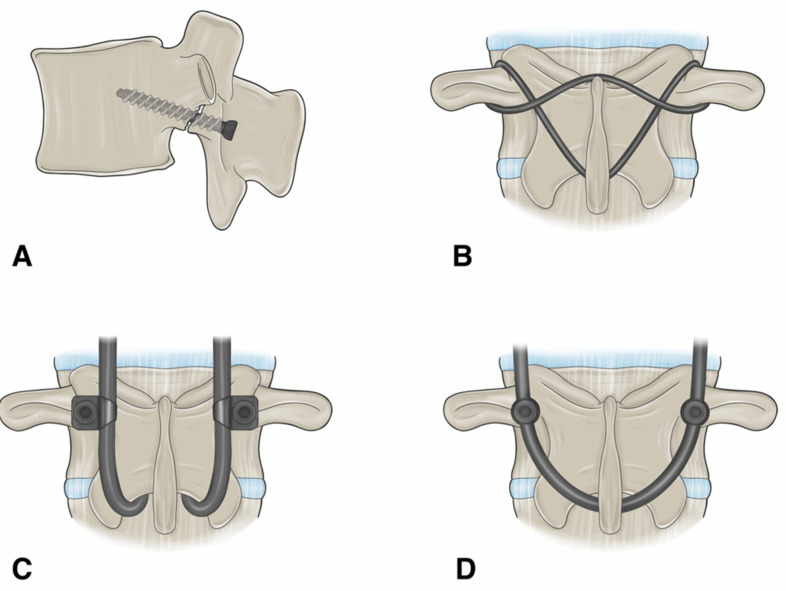
An illustration showing the different surgical techniques for direct pars repair. (**A**) Buck’s repair technique. A screw is passed across the pars defect. (**B**) Scott’s repair technique. The wire is passed around the transverse process and spinous process. (**C**) Morscher repair technique using pedicle screw with laminar hooks. (**D**) Pedicle screw-based technique with a U-shaped rod. “Used with permission from The University of Miami Department of Neurological Surgery, Miami, FL, USA”.

**Figure 4:**
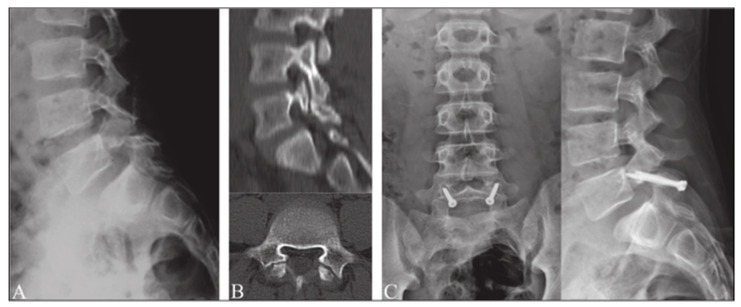
Buck’s repair technique. (**A**) Preoperative radiograph in lateral projection. (**B**) CT scan of pars defect. (**C**) Postoperative radiographs after surgery using Buck’s repair technique. “Reproduced with permission from Sri Vijay Anand, K.S.; Eamani, N.; Shetty, A.; Rajasekaran, S. Spondylolysis and pars repair technique: A comprehensive literature review of the current concepts. *Indian Spine J.* **2021**, *4*, 29” [38].

The technique described by Buck in 1970 involved the internal reduction of the fracture by inserting a single cortical screw across the pars defect after placing the supporting autologous graft into the defect (Figure 3A and Figure 4) [37,38]. After utilizing this direct repair technique on a total of 16 patients, Buck reported only one failure, which was described as the loosening of both screws and an unstable lamina one year after the procedure was performed. Two patients developed complications of post-operative sciatica, one of which was developed due to the misplacement of a screw. Buck provided one of the first descriptions of bilateral screw fixation across the pars interarticularis defect [39]. Advantages of Buck’s technique include preservation of segment motion, rapid postoperative recovery, and minimal blood loss [40]. Further studies detailing utilization of Buck’s technique demonstrated satisfactory results in 78–90% of patients [41]. In 1988, Pederson performed a direct repair using Buck’s technique to treat 18 patients with either symptomatic spondylolysis or Grade-I spondylolisthesis, who showed no improvement after non-operative treatment measures. The result was satisfactory in 83% of patients: “excellent” in 10 patients, “good” in five, and “poor” in three [42]. In 1991, Bonnici et al. investigated the utilization of the Buck technique to perform posterolateral screw fixation on 24 patients with painful isthmic spondylolysis and up to Grade-I spondylolisthesis in the lumbar spine [43]. An average age of 29 years was reported with an average follow-up period of 5 years, ranging from 13 months to 12 years. The surgery was performed on individuals with persistent disabling lower back pain. The results of the study revealed a total of 21 patients with complete pain relief or an occasional complaint of backache or discomfort. Only two of the 24 patients were satisfied with the surgery and rated the results as excellent or good. The findings of this investigation demonstrated Buck screw fixation technique as being a safe and reliable treatment method for painful Grade-I spondylolisthesis secondary to isthmic spondylolysis in young active adults with a low complication rate [43]. In 2014, Snyder et al. identified 16 patients who were treated with Buck’s operation from 2004 to 2010. The mean age of the patients was 16 years, with 14 being 20 years or younger at the time of treatment. Of the 16 patients, 100% had axial back and 38% had concomitant radiculopathy. A total of 29 pars defects were treated using Buck’s technique with 81% of patients having bilateral pars defects and 19% unilateral. Complete or partial symptom relief was noted in 15 patients (94%). Healing was reported in 26 of the 29 pars defects (89.6%) prior to 1 revision surgery and last radiological follow-up revealed an overall fusion rate of 97% [44]. A recent review of surgical techniques by Debnath revealed Buck’s direct repair technique to be the most reliable, safe, and reproducible method with the best clinical outcome [19]. The main challenge with Buck’s technique was reported as being the technical difficulty related to placement of screws as there is little room for error [19].

#### 1.9.2. Morscher Screw-Hook Repair

In 1984, Morscher noticed several disadvantages of Buck’s repair, inspiring him to create his own method of fixing pars defects. Morscher noted that it was extremely difficult to place screws in the neural arch in patients with dysplasia [45]. He described how the lack of good fixation and inadequate bone for fusion often led to the screws breaking in a high percentage of his cases, coinciding with poorer patient outcomes [45]. These issues led to the development of the hook-screw repair technique (Figure 3C).

Originally, the hook used was a Harrington-type hook with a narrow slot to better fit the vertebral arch [45]. It was available in 6 mm and 8 mm sizes, and had a spur located inside the slot to prevent slippage [45]. The screw passes through the hook into the superior articular process and obtains a 4 mm cancellous thread [45]. The opposite end of the screw is triangular to accommodate a special wrench for insertion [45]. The screws are available in 40 mm, 50 mm, 55 mm, 60 mm lengths [45]. 

**Figure 5:**
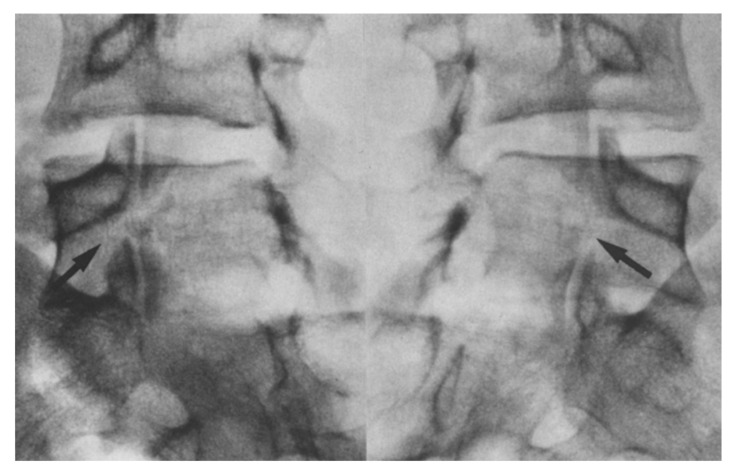
Oblique radiographs showing a bilateral pars defect in the L5 of a 17 year old patient [45]. The arrows depict the specific sites of the defect. “Used with permission of Springer Nature BV, from Hefti, F.; Seelig, W.; Morscher, E. Repair of lumbar spondylolysis with a hook-screw. *Int. Orthop.* **1992**, *16*, 81–85; permission conveyed through Copyright Clearance Center, Inc.”.

**Figure 6:**
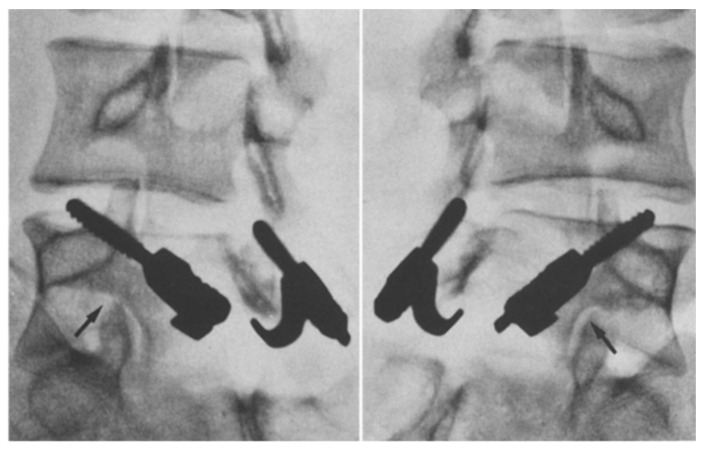
Radiographs showing solid fusion of L5 in the same patient one-year post-op [45]. The arrows depict the site of fusion. “Used with permission of Springer Nature BV, from Hefti, F.; Seelig, W.; Morscher, E. Repair of lumbar spondylolysis with a hook-screw. *Int. Orthop.* **1992**, *16*, 81–85; permission conveyed through Copyright Clearance Center, Inc.”.

The operation starts with exposing the vertebral arches from L4-S1, clearing surrounding connective tissue, and roughening the adjacent bone in the process [45]. After exposure, the lower end of the inferior articular process at L4 is cut while the ligamentum flavum is cleared from the inferior edge of the arch of L5 [45]. A small notch is then created in the inferior aspect of the L5 vertebral arch, and the hook is placed on the arch in such a way that the opening for the screw is directed toward the base of the superior articular process [45]. Next, a 2.5 mm drill is driven into the base of the articular process outside of the spondylitic effect, so that the entire base of the articular process is penetrated [45]. Cancellous bone from the iliac crest is then placed in the lateral aspect of the defect, and the screw is passed through the hook and bone [45]. Finally, two nuts are placed on the screws for compression and projecting portion of the screw is excised (Figure 5 and Figure 6) [45].

Mohammed et al. analyzed the efficacy and outcomes of multiple repair methods for pars fractures. Five studies with a total of 193 patients who received the hook-screw repair method for their pars defect were assessed [3]. The authors found that fusion was obtained in 77.72% of these patients, which was the lowest rate of the four techniques analyzed (Scott’s technique, Buck’s technique, pedicle screw technique, and Morscher’s technique) [3]. Morscher’s method resulted in highest complication rate at 27.42%, with common complications being root irritation, implant loosening, and pseudoarthrosis [3].

#### 1.9.3. Scott’s Wiring Technique

In 1986, Nicol and Scott reported the method of direct repair using cerclage wire fixation. The technique involves a wire being passed around the transverse and spinous processes along with bone grafting (Figure 3B) [3,46]. The report evaluated the results in seven patients with low back pain who failed conservative treatment, and included follow-up ranging from 2 to 12 years. At follow-up, all patients except for one showed healed lesions with intact wires and were without spondylolisthesis. The only known operative failure involved a patient with persistent symptoms who was found to have wire breakage, demonstrating nonunion. The advantages of this technique related to its simplicity and the use of wire leaving the entire bony surface of each side of the defect free to participate in bony union [3,46]. Johnson and Thompson utilized the Scott technique between 1979 and 1989 to treat 22 patients with symptomatic lumbar that had not responded to conservative treatment. The mean age of patients was 15 years and mean follow-up was four years. Of the 19 patients under 25 years old, all had satisfactory results. Poor results were reported in two of the three patients under the age of 25 [47]. In 2014, a retrospective study by Hioki et al. evaluated clinical outcomes of bony union in 44 athletes with symptomatic spondylolysis after undergoing segmental wiring fixation. The results revealed bilateral bony union in 29 cases (67.4%), unilateral union in six cases (13%), and nonunion in nine cases (19.6%) [48]. Disadvantages of the Scott technique have been reported as wire breaking, nerve root injury, muscular dissection, and less stiffness [19,27,46]. A 2022 meta-analysis by Tsai et al. demonstrated that Scott repair technique has a higher complication rate and low union rates when compared with other surgical techniques [49].

#### 1.9.4. Pedicle Screw Based Repairs

In 1996, Tokuhashi and Matsuzaki reported the results of utilizing segmental pedicular screw hook fixation on six male patients with lumbar spondylolysis. The age of patients ranged from 17 to 32 years and the mean follow-up period was 29.8 months. Postoperative results showed that all patients had significant relief in their low back pain or radicular pain. There were no complications or instrumentation failures observed with this approach. Advantages of segmental pedicular screw hook fixation were described as the ability to graft a large bone within the defect and the lack of dependence on shape of the defect [50]. Pedicle screw hook fixation has been described as an evolution of Morscher’s technique (Figure 3D) [51]. Kakiuchi detailed the use of pedicle screws and laminar hooks for durable fixation of a pars interarticularis defect in a 1997 report. A total of 16 patients underwent this procedure after failed non-operative management of their bilateral pars defects with or without Grade I or II spondylolisthesis. The average age at the time of operation was 32 years and average postoperative follow-up was 25 months. Osseous union of bilateral pars defects was observed in oblique radiographs of all 16 patients and relief of symptoms was seen in 13 patients. There were no complications reported. Overall, this use of pedicle screws, rods, and laminar hooks with bone graft was described as a simple and effective technique for repair of pars interarticularis defects [52]. In 1988, Songer and Rovin reported preliminary findings of pars interarticularis reconstruction with cable-screw construct on seven patients who had low back pain that did not respond to conservative treatment and Grade I or less spondylolisthesis. With this technique, a special pedicle cable-screw was placed into the pedicle of the vertebra, followed by passing of a double cable under the lamina, threaded through the pedicle screw, and wrapped around the spinous process. The mean age of patients was 20.5 years, and the average follow-up period was 25.5 months. The duration of symptoms before surgery was an average of 31 months. A total of five patients rated the results as “excellent” and two rated results as “good” as per the Prolo score. Plain radiographs of the lumbar spine revealed union of the pars interarticularis in all seven patients [53].

In 2017, a retrospective review by Raudenbush et al. described the results of nine adolescent patients treated with indirect pars repair using pedicle screws, laminar hooks, and local autograft for spondylolysis with or without low-grade spondylolisthesis. The average length of follow-up was 11.9 months. Results revealed a decrease in the average VAS preoperatively (5.6 points) to final follow-up (1.2). Postoperative radiographs demonstrated definitive bony healing in seven of the nine patients [54]. Recently, a 2022 report by Gao et al. described the application of the pedicle screw-rod-hook technique for direct pars repair in 64 adult patients with low-grade isthmic spondylolisthesis. The average follow-up was 52.15 months. The results demonstrated significant reduction in visual analog scale (VAS) scores and Owestry Disability Index (ODI) postoperatively. According to the modified Prolo score, 60 patients (93.75%) were “excellent” and 4 patients (6.25%) were “good” A success rate of 96.86% was reported for successful bony fusion [55]. In 2022, Linton and Hsu described challenges associated with the pedicle-screw hook technique as the difficult screw placement on abnormal lamina and the application of enough compression to approximate the pars defect [27]. Cadaveric studies provided biomechanical data that illustrate the pedicle-screw technique as providing the greatest flexion/extension stability. Higher fusion rates and lower complication rates have made the pedicle screw approach the preferred method for direct repair [27,56].

### 1.10. Minimally Invasive Surgical Techniques

The growing interest in minimally invasive surgery has led to the development of modified techniques for repair of pars interarticularis defects. Advantages of minimally invasive surgical approach include minimized blood loss, lower complication rates, shorter hospital stays, and decreased evidence of surgical site infection [57,58]. Multiple studies that employed minimally invasive approaches have demonstrated low complication rates and excellent outcomes [3]. Our aim was to identify literature that illustrates the various uses of minimally invasive techniques for direct pars repair.

In 1999, Gillet and Petit provided a description of a minimally invasive technique that utilized a V-shaped rod and pedicle screws along with bone grafting of pars defect. The procedure was performed on 10 patients with pars defect that did not respond to conservative treatment. The patients had a mean age of 26 years at time of operation and an average postoperative follow-up of 35 months. The results revealed an “excellent” outcome in 6 patients, “good” in one patient, and “fair” in one patient. There was one reported failure of the procedure. There were no complications reported [59]. This technique has become known as the “smiley face” rod method, as the screw head and rod resembled a smiley face.

In 2006, Ulibarri et al. compared biomechanical properties of direct pars repair using an intralaminar link construct to spondylolysis repair constructs that were previously established. The novel technique involved the placement of multiaxial pedicle screws and a modular linkage that passed beneath the spinous process of the same segment. Results of the biomechanical comparison revealed the least displacement across pars defect with the intralaminar link construct and had both the highest interbody flexion and extension stiffness and the highest interbody torsional stiffness with the pedicle screw-rod-hook technique. A retrospective study of five patients who underwent this procedure was also carried out and the average follow-up was 4.6 years. The results showed that all five patients had complete healing, as observed by radiography, or pain relief. One patient with solid healing later had pain that was resolved with removal of the implant [60]. A description of a minimally invasive approach to direct pars repair without requiring fusion across a motion segment was provided by Gillis et al. in 2015. The surgical approach was similar to a modified Scott technique and involved the placement of a “sandwich” of corticocancellous bone inside a sponge of bone morphogenic protein (BMP) into the defect to promote fusion. A total of eight competitive athletes with spondylolysis without spondylolisthesis underwent this minimally invasive procedure for bilateral direct pars defect repair. The age of patients ranged from 16 to 23 years. Results revealed six patients returned to their previous level of competitiveness and two failed fusions of spondylolysis. There was a mean improvement in patient reported outcome measures and all patients reported clinical improvement postoperatively [61].

In 2019, Fayed et al. published a case series involving two patients that underwent minimally invasive direct pars repair via percutaneous insertion of cannulated lag screw. The screw was inserted across the pars defect with compression against the lamina. The first patient was 22 years old, and the second patient was 23 years old with follow-up periods of 25 months and 21 months, respectively. Successful fusion was seen in the first patient while hardware failure was observed in the second patient, requiring subsequent revision. Additionally, fracture morphology differed between the two patients with the first patient having linear fractures oriented perpendicular to screw trajectory and the second patient having a curved defect. The results demonstrated the potential role of pars fracture morphology and orientation in nonunion [57]. In 2020, Tian et al. investigated the use of a novel robotic system for intralaminar screw fixation of spondylolysis. Direct intralaminar screw fixation with TiRobot system guidance was performed on a 26-year-old patient with bilateral spondylolysis. The outcomes revealed safe and accurate screw placement, confirmed by CT, and no intraoperative complications [62]. A month later, Takeuchi et al. shared a case report on the use of the double “smiley face” rod method to treat double-level spondylolysis. The 29-year-old patient underwent direct repair of bilateral pars defects at L4 and L5 using this surgical approach at both vertebral levels. There were no complications and clinical improvement was reported one year after the surgery [63]. In 2021, Yurac et al. performed a modified Buck technique repair with a minimally invasive technique involving cannulated compression screws along with neuronavigation and neuromonitoring. A total of three athletes, between 17 and 18 years old, with bilateral spondylolysis underwent this procedure. They were followed for a minimum of three months and the outcomes revealed all patients returned to their sports activities in less than six months. There were no complications reported [64].

Recently, a 2023 case report by Tatsumura et al. described a modified smiley face rod technique for pars defect repair. This method preserved the erector spinae muscles through placement of pedicle screws in the lateral edge of the pedicle. The supraspinous ligament was also preserved via insertion of a U-shaped rod between the spinous processes. The case report details utilization of this technique to repair pseudarthrotic lumbar spondylolysis. One year postoperatively, fusion of the pars defect was demonstrated by CT [65]. In this same year, a retrospective cohort study by Zhang et al. investigated the clinical efficacy of minimally invasive transforaminal lumbar interbody fusion (MIS-TLIF) to treat II° lumbar isthmic spondylolisthesis (IS). This method involves the intermuscular approach, causing less damage than the open approach and a faster recovery. Of the 101 patients with II° lumbar IS, 53 underwent MIS-TLIF surgery and 48 underwent open transforaminal lumbar interbody fusion (OPEN-TLIF) surgery. The results revealed a significantly lower intraoperative blood loss, postoperative drainage, and postoperative hospital stay in those who underwent MIS-TLIF as compared to those who underwent OPEN-TLIF. No significant differences regarding postoperative leg pain score, slippage rate, and fusion rate were noted [66].

#### Levi Technique—Minimally Invasive Direct Pars Screw Placement

In 2013, Widi, Williams, and Levi reported on the technique of lumbar spondylolysis repair using a minimally invasive approach, a modification of the Buck technique, to place pars interarticularis screws with an intraoperative imaging. Biplanar fluoroscopy was used to determine trajectory of the pars screw and an intraoperative CT scanner was used to confirm proper placement of the guide wire and the screws which was supplemented with local autograft and bone morphogenetic protein at the fracture site. The procedure was performed on three young athletic patients, 17–25 years old, with a bilateral pars defect that did not heal with conservative management. There was satisfactory screw placement with evidence of fusion at the pars repair at 12 months [67]. Further evaluation of this technique on a larger number of patients with longer follow-up was published in 2017 by the University of Miami group which included Ghobrial, Levi, and additional authors. The report described minimally invasive direct pars repair with the use of cannulated screws and recombinant human bone morphogenetic protein. A total of nine patients, 14-25 years old, underwent this surgery to treat lumbar spondylolysis. Bilateral pars fractures of L4 or L5 were observed in all nine patients and the duration of preoperative symptoms ranged from 9 to 48 months. The operative duration was an average of 189 min, and the mean intraoperative blood loss was reported as 17.5 mL. The average follow-up period was 30.8 months and there were no intraoperative complications reported. All six patients with more than one year follow-up had successful fusion as evident on CT and radiographic imaging. Asymptomatic unilateral screw backout was noted in one patient [68].

**Figure 7:**
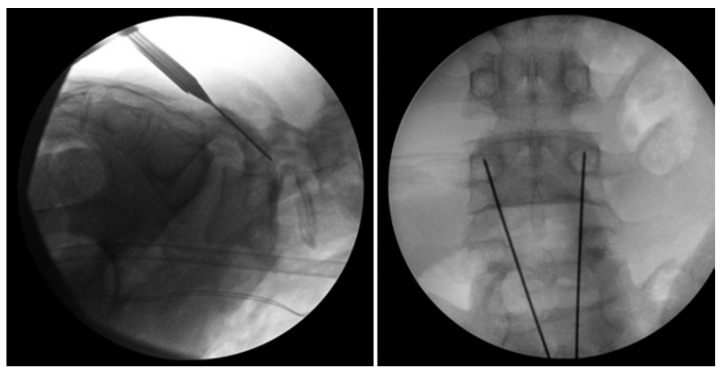
Bilateral L5 intralaminar threaded guidewire placement in the lateral (**left**) and AP (**right**) projections demonstrated by intraoperative fluoroscopic views. Lateral laminar entry and bisect the pedicle is demonstrated by the guidewires on the AP projection. The lateral projection shows appropriate clearance of the L5–S1 neural foramen, and the rostral endplate or foramen is not violated [68]. “Reproduced with permission from Ghobrial, G.M.; Crandall, K.M.; Lau, A.; Williams, S.K.; Levi, A.D. Minimally invasive direct pars repair with cannulated screws and recombinant human bone morphogenetic protein: case series and review of the literature; published by Neurosurg. *Focus* **2017**, *43*, E6”.

**Figure 8:**
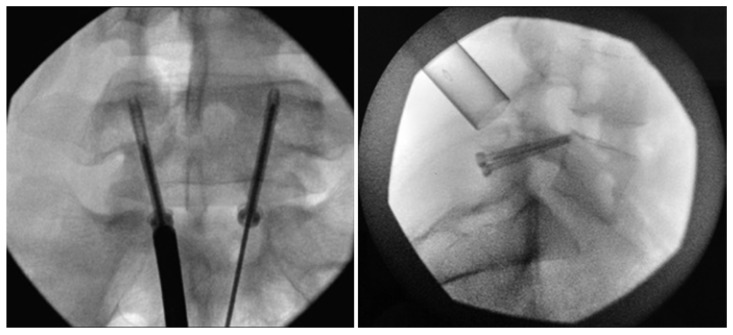
Bilateral placement of cannulated pars screws over threaded guidewires (**left**) and subsequent bilateral exposure of the pars fracture demonstrated by intraoperative fluoroscopic views in the AP (**left**) and lateral (**right**) projection. Tubular-based retractors (**right**) provide visualization of pars fracture for decortication [68]. “Reproduced with permission from Ghobrial, G.M.; Crandall, K.M.; Lau, A.; Williams, S.K.; Levi, A.D. Minimally invasive direct pars repair with cannulated screws and recombinant human bone morphogenetic protein: case series and review of the literature; published by Neurosurg. *Focus* **2017**, *43*, E6”.

An in-depth description of the minimally invasive direct pars screw placement was provided by Raffa, Luther, and Levi in 2019. Fluoroscopy in the AP and lateral planes (Figure 7 and Figure 8) is used to calculate the ideal pars screw trajectory, which should transverse the middle of the pars. Fluoroscopic guidance is also utilized for tunneling of the cannula through soft tissues before being docked onto the inferior surface of the lamina. Additional imaging via an intraoperative CT scan is later performed to confirm proper positioning of guide wires. Prior to final screw tightening, rhBMP and bone shavings are placed into each defect [5,67,68].

**Figure 9:**
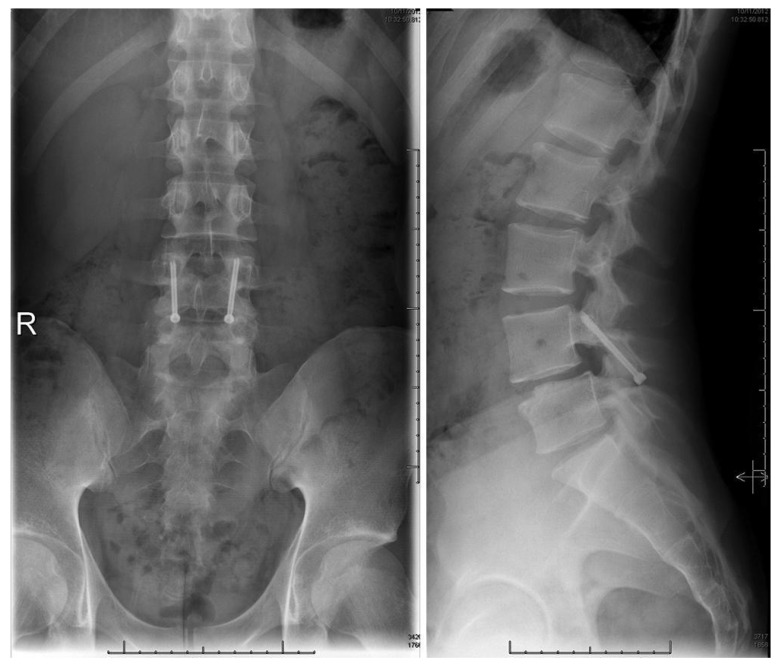
Postoperative upright standing radiographs in AP (**left**) and lateral (**right**) projections demonstrating bilateral placement of lag screws [68]. “Reproduced with permission from Ghobrial, G.M.; Crandall, K.M.; Lau, A.; Williams, S.K.; Levi, A.D. Minimally invasive direct pars repair with cannulated screws and recombinant human bone morphogenetic protein: case series and review of the literature; published by Neurosurg. *Focus* **2017**, *43*, E6”.

**Figure 10:**
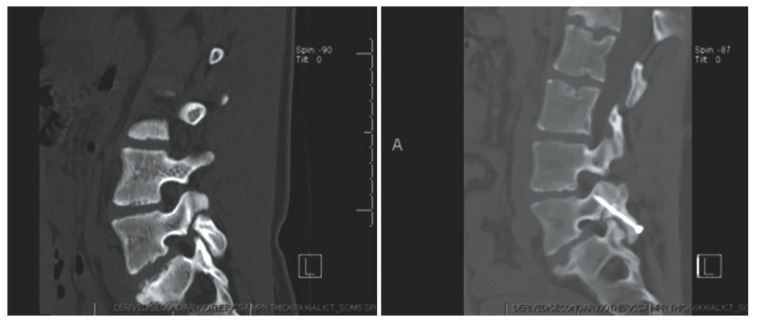
Case 8. Chronic L-4 pars defect in a 15-year-old girl. Sagittal CT of the lumbar spine, illustrating left L-4 pars fracture (**left**). CT obtained at 3 months follow up demonstrating fusion across the fracture (**right**). “Reproduced with permission from Ghobrial, G.M.; Crandall, K.M.; Lau, A.; Williams, S.K.; Levi, A.D. Minimally invasive direct pars repair with cannulated screws and recombinant human bone morphogenetic protein: case series and review of the literature; published by Neurosurg. *Focus* **2017**, *43*, E6”.

An update on the Levi technique of pars interarticularis screw placement via minimally invasive techniques utilizing intraoperative biplanar fluoroscopy in 24 young adult athletes is provided in this review. All patients participated in sports including, though not limited to hockey, ballet, basketball, football, etc. The mean age [±SD] of patients was 17.4 ± 3.1 years (range 13–25 years) with 18 (75%) being male. The average duration [±SD] of preoperative symptoms was 10 ± 5.6 months. All patients presented with pars fractures at the L2 (*n* = 1), L4 (*n* = 8), or L5 (*n* = 16) vertebral level. One patient had a pars fracture at both L4 and L5 level. Another patient presented with a fracture of the left pars and right pedicle in the L5 vertebra. All patients underwent a minimum of six months of conservative therapies, although most went through at least one year of conservative treatment. Nonoperative management included three months of sports cessation and bracing early on if the patients presented with an acute fracture. This was often followed by physical therapy and medication management consisting of anti-inflammatories, muscle relaxants, and epidural injections into the specific pars defect. The mean duration of postoperative follow-up [±SD] was 35.4 months ± 34.8 months (Figure 9 and Figure 10). One patient was found to have new fractures at L2 and L3 upon follow-up unrelated to the original surgical procedure. Screws were removed from another patient, 24 months after the operation due to concerns for their contribution to the patient’s back pain. There was one case of pseudoarthrosis and lucencies around the screws at 12 months in a patient with heavy nicotine exposure. Screws were removed in 2 patients—one patient who had fused but there was concern that the screws were possibly causing back pain and another in which there was evidence of fusion at fracture site but lucencies around the screws and concern for possible infection—but work-up for infection was negative. The overall fusion rate was 95.8%. Challenges were occasionally faced when performing this operation in patients aged 13 to 14, due to their rapid growth and small bony anatomy.

## 2. Conclusions

Pars interarticularis fractures are posterior vertebral arch defects that occur between the superior and inferior articular facets [5,7,8,10,69]. After failed conservative and medical management of the resulting lumbar back pain, the next best treatment option is surgical repair [5,9,16,37]. In this review, we describe several conventional surgical techniques while highlighting the benefit of minimally invasive methods for repairing pars defects. Furthermore, we discuss the Levi technique as a minimally invasive method for repairing pars fractures. Overall, this injury is prevalent in society, and updated literature regarding surgical repair is important in continuing to produce positive outcomes for patients.
